# 3D-structured illumination microscopy reveals clustered DNA double-strand break formation in widespread γH2AX foci after high LET heavy-ion particle radiation

**DOI:** 10.18632/oncotarget.22679

**Published:** 2017-11-25

**Authors:** Yoshihiko Hagiwara, Atsuko Niimi, Mayu Isono, Motohiro Yamauchi, Takaaki Yasuhara, Siripan Limsirichaikul, Takahiro Oike, Hiro Sato, Kathryn D. Held, Takashi Nakano, Atsushi Shibata

**Affiliations:** ^1^ Education and Research Support Center (ERSC), Gunma University, Maebashi 371-8511, Japan; ^2^ Department of Radiation Oncology, Gunma University Graduate School of Medicine, Maebashi 371-8511, Japan; ^3^ Research Program for Heavy Ion Therapy, Division of Integrated Oncology Research, Gunma University Initiative for Advanced Research (GIAR), Maebashi 371-8511, Japan; ^4^ Department of Molecular Metabolic Regulation Research, Sasaki Institute, Tokyo 101-0062, Japan; ^5^ Department of Radiation Biology and Protection, Atomic Bomb Disease Institute, Nagasaki University, Nagasaki 852-8523, Japan; ^6^ Laboratory of Molecular Radiology, Center for Disease Biology and Integrative Medicine, Graduate School of Medicine, The University of Tokyo, Tokyo 113-8655, Japan; ^7^ Faculty of Pharmacy, Silpakorn University, Nakhon Pathom 73000, Thailand; ^8^ Department of Radiation Oncology, Massachusetts General Hospital/Harvard Medical School, Boston, MA 02114, USA; ^9^ International Open Laboratory, Gunma University Initiative for Advanced Research (GIAR), Gunma 371-8511, Japan

**Keywords:** clustered DNA double strand break, heavy-ion radiation, carbon-ion therapy, γH2AX foci, RPA foci

## Abstract

DNA double-strand breaks (DSBs) induced by ionising radiation are considered the major cause of genotoxic mutations and cell death. While DSBs are dispersed throughout chromatin after X-rays or γ-irradiation, multiple types of DNA damage including DSBs, single-strand breaks and base damage can be generated within 1–2 helical DNA turns, defined as a complex DNA lesion, after high Linear Energy Transfer (LET) particle irradiation. In addition to the formation of complex DNA lesions, recent evidence suggests that multiple DSBs can be closely generated along the tracks of high LET particle irradiation. Herein, by using three dimensional (3D)-structured illumination microscopy, we identified the formation of 3D widespread γH2AX foci after high LET carbon-ion irradiation. The large γH2AX foci in G_2_-phase cells encompassed multiple foci of replication protein A (RPA), a marker of DSBs undergoing resection during homologous recombination. Furthermore, we demonstrated by 3D analysis that the distance between two individual RPA foci within γH2AX foci was approximately 700 nm. Together, our findings suggest that high LET heavy-ion particles induce clustered DSB formation on a scale of approximately 1 μm^3^. These closely localised DSBs are considered to be a risk for the formation of chromosomal rearrangement after heavy-ion irradiation.

## INTRODUCTION

Ionising irradiation (IR) is a major source of DNA double-strand breaks (DSBs). DSBs are considered the most critical DNA lesion because they lead to cell death if unrepaired and cause deleterious mutations if misrepaired [1]. X-rays or γ-irradiations induces DSBs with a random distribution in the nucleus because of their low-density energy deposition, whereas heavy-ion particle radiation,which has a high linear energy transfer (LET), deposits its energy densely along the track of particle traversal causing non-random distribution of DSBs [2]. It is well accepted that high LET particle radiation induces strong cell-killing effects compared with those after X-rays or γ-irradiations [3]. By exploiting this property, particle radiation can be applied in cancer therapy [4]. For instance, Carbon-ion (C-ion) irradiation, which is categorised as heavy-ions, shows a 2–3-fold greater relative biological effectiveness (RBE) for killing cells than X-rays or γ-irradiations [5–8]. In addition, because heavy-ion particle radiation produces a highly concentrated dose distribution because of the Bragg peak effect, it has a great advantage to intensively target cancer cells and minimalize cellular damage to the surrounding normal tissues. Accumulating studies have demonstrated that heavy-ion radiation causes a greater number of dynamic chromosomal aberrations, such as chromosomal rearrangements including dicentric, acentric, translocation and deletion mutations, other than X-rays [9–11]. Such dynamic chromosomal aberrations are considered a cause of the strong cell-killing effect after heavy-ion irradiation, particularly when dicentrics are generated or large deletions are caused in essential genes. In addition to the dynamic chromosomal aberrations, biological analyses and Monte Carlo simulations suggest that heavy-ion radiation induces complex DNA lesions, defined as DNA damage containing both DSBs and single-strand breaks (SSBs), as well as base damage, within 1–2 helical turns (10–20 nm) [12–14]. These complex lesions are considered unrepairable, or they delay the speed of overall DNA repair, which may result in misrepair [12, 15, 16]. These complex lesions are hallmarks of DNA damage after high LET heavy-ion particle radiation. However, it has been under debate whether they are the direct causes of rearrangement at the chromosomal level.

DSBs are repaired by either non-homologous end joining (NHEJ) or by homologous recombination (HR) after IR. In the G_1_-phase, DSBs are mainly repaired by NHEJ after X-rays or C-ion irradiation [17, 18], whereas in the G_2_-phase, some DSBs are repaired by HR, while the majority are repaired by NHEJ after X-rays [16]. However, importantly, DSBs are preferentially repaired by HR in the G_2_-phase after heavy-ion particle irradiation [16]. IR activates DNA damage signals, which are required for DNA repair and cell cycle checkpoint arrest [1]. ATM and DNA-PKcs are immediately activated at DSB sites where they phosphorylate H2AX, a variant form of the histone H2A, around DSBs [19]. The phosphorylated form of H2AX (γH2AX) has been well utilised as a marker of DSBs because γH2AX is visualized as defined and countable foci using microscopy, particularly when <100 foci are induced after IR [20]. In contrast to γH2AX foci after X-rays, large γH2AX foci have been frequently observed along the particle track after high LET heavy-ion irradiation [17, 21]. In recent years, technology advances in immunofluorescence microscopy have substantially improved the resolution of this visualization technique. Super-resolution microscopy, such as three dimensional structured illumination (3D-SIM), stimulated emission depletion (STED) and photo-activated localisation microscopy (PALM) have achieved <50–100 nanometer resolution [22]. We previously demonstrated, using a DeltaVision microscope with deconvolution, that after heavy-ion irradiation, the large foci of γH2AX or 53BP1, which functions downstream of γH2AX and is a marker of DSBs, encompass multiple and discrete smaller foci [17, 23]. This observation suggests that multiple DSBs are likely generated in close proximity along the track of high LET heavy-ion radiation. However, because H2AX phosphorylation, followed by 53BP1 recruitment, occurs around DSBs throughout thousand- to mega-base-pairs of chromatin [20], the γH2AX or 53BP1 signal is unlikely to represent the position of a DSB when super-resolution microscopy is used. Rather, the foci represent an area of DNA damage-dependent signal expansion on the chromatin around DSBs [24]. Most recently, several approaches were applied to visualize the clustered DSB formation after high LET irradiation, supporting the notion that high LET heavy-ion induce multiple DSBs in close proximity along the particle track [25–27], although the distribution of DSBs has not been fully analysed three-dimensionally yet.

In this study, we applied the 3D-SIM technique to investigate three-dimensional distribution of DNA damage signals induced by heavy-ion irradiation in whole cells, and we examined the 3D volume of γH2AX foci after C-ion irradiation. Because we aimed to investigate the distribution of DNA damage in the therapeutic dose range, we used 20 or 60 keV/μm C-ion irradiation in this study. Our results indicate that the volume of γH2AX foci after C-ion irradiation was significantly greater than that observed with X-rays. Interestingly, the large γH2AX foci in G_2_-phase cells encompassed multiple replication protein A (RPA) foci, which is a marker of DSBs undergoing resection during HR, whereas such clustered RPA foci were rarely observed after X-rays. In addition, the volume of γH2AX foci was correlated with the number of RPA foci, suggesting that formation of large γH2AX foci is caused by multiple DSBs formed along the track. Finally, 3D-SIM analysis showed that any two individual RPA foci within the γH2AX foci are separated by an average distance of 700 nm. Collectively, this is the first study to demonstrate three-dimensional distribution of clustered DSBs in close proximity on the order of several hundred cubic nanometres after high LET heavy-ion irradiation.

## RESULTS

### 3D-SIM analysis revealed that C-ion irradiation causes widespread γH2AX foci along the particle track

**Figure 1:**
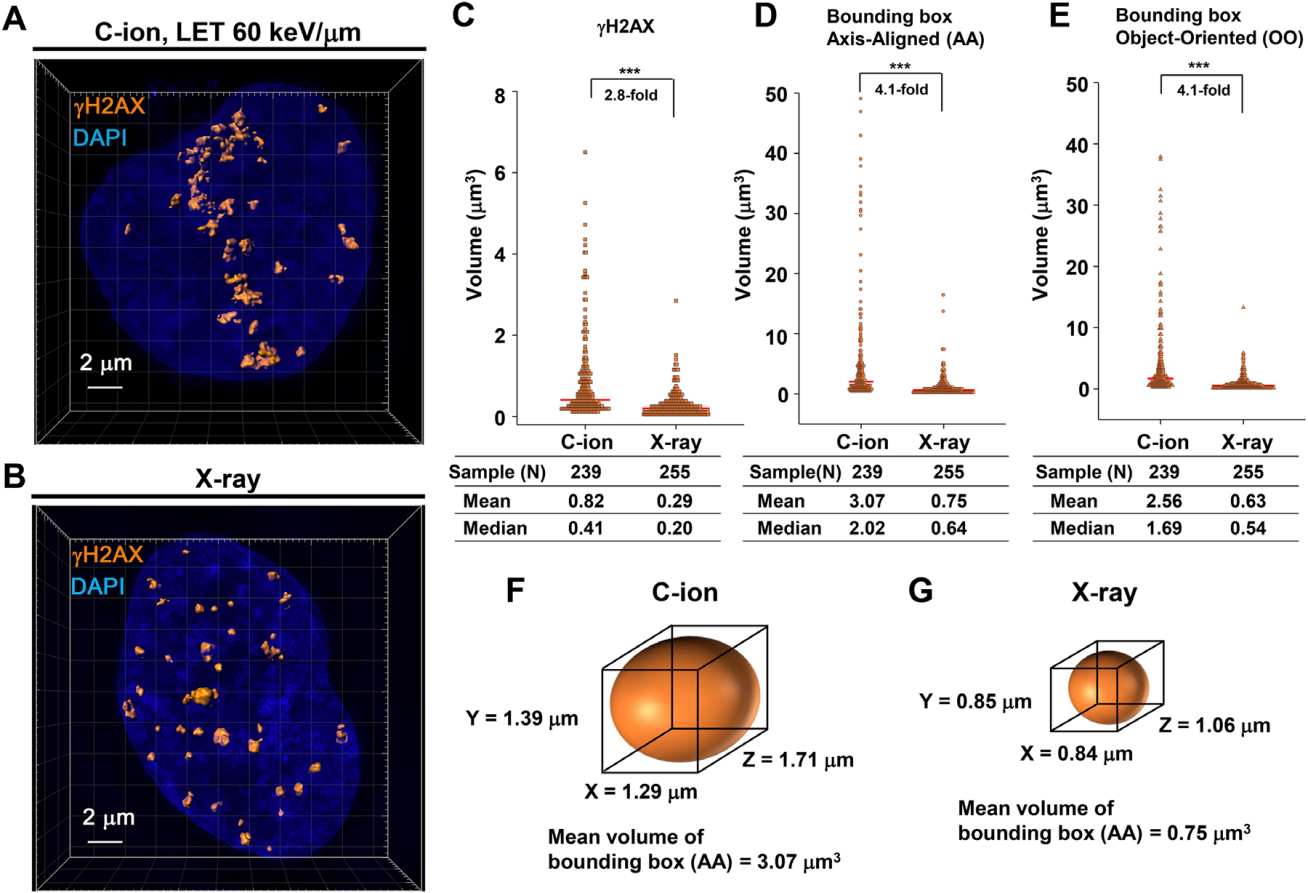
3D-SIM analysis revealed that C-ion irradiation causes widespread γH2AX foci along the particle track **(A, B)** 1BR hTERT cells were irradiated with 1 Gy C-ion irradiation with LET 60 keV/μm (A) or with 100 kVp X-rays (B). Cells were fixed 30 min after irradiation and stained with γH2AX and DAPI. The surface polygon images of γH2AX foci were generated by Imaris 8.1.2. **(C-E)** The density dot plots of the volume of γH2AX foci (C) and bounding box Axis-Aligned (AA) (D) and bounding box Object-Oriented (OO) (E) around γH2AX foci in 1BR hTERT cells are shown. **(F, G)** Representative volume of bounding box AA around γH2AX foci after C-ion irradiation (F) or X-rays (G) in 1BR hTERT cells is shown.

To investigate the magnitude of 3D γH2AX foci expansion after high LET heavy-ion radiation, IR-induced γH2AX foci were analysed using super-resolution modes of a 3D-SIM in the DeltaVision OMX system (Figure 1A, 1B; Note: to examine RPA foci within γH2AX foci thereafter, we examined cells in G_2_-phase (CENPF+) in this study (Supplementary Figure 1). In this study, we used 1BR hTERT human fibroblasts unless stated otherwise. γH2AX foci volume was measured following the generation of polygon rendering by the imaging software Imaris 8.1.2 (Figure 1A, 1B, Supplementary Figure 2 and Supplementary Figure 3). Consistent with the notion obtained by lower resolution microscopy [17, 21], the volume of γH2AX foci after C-ion irradiation was 2.8-fold larger than that observed with X-rays at 30 min after 1 Gy (Figure 1C). In addition, to analyse the distribution of the 3D foci spread, we measured the volume and length of the x, y and z-axis of a bounding box Axis-Aligned (AA) and Object-Oriented (OO) around γH2AX foci (Figure 1D-1G and Supplementary Figure 4). Similar to the actual γH2AX foci volume, the volume of bounding boxes AA and OO was approximately 4-fold larger than those observed after X-rays, suggesting that 3D γH2AX foci after C-ion irradiation have a greater spread than after X-rays (Figure 1C-1G).

### 3D-SIM analysis revealed clustered RPA foci formation in γH2AX foci in G_2_-phase cells after C-ion irradiation

**Figure 2:**
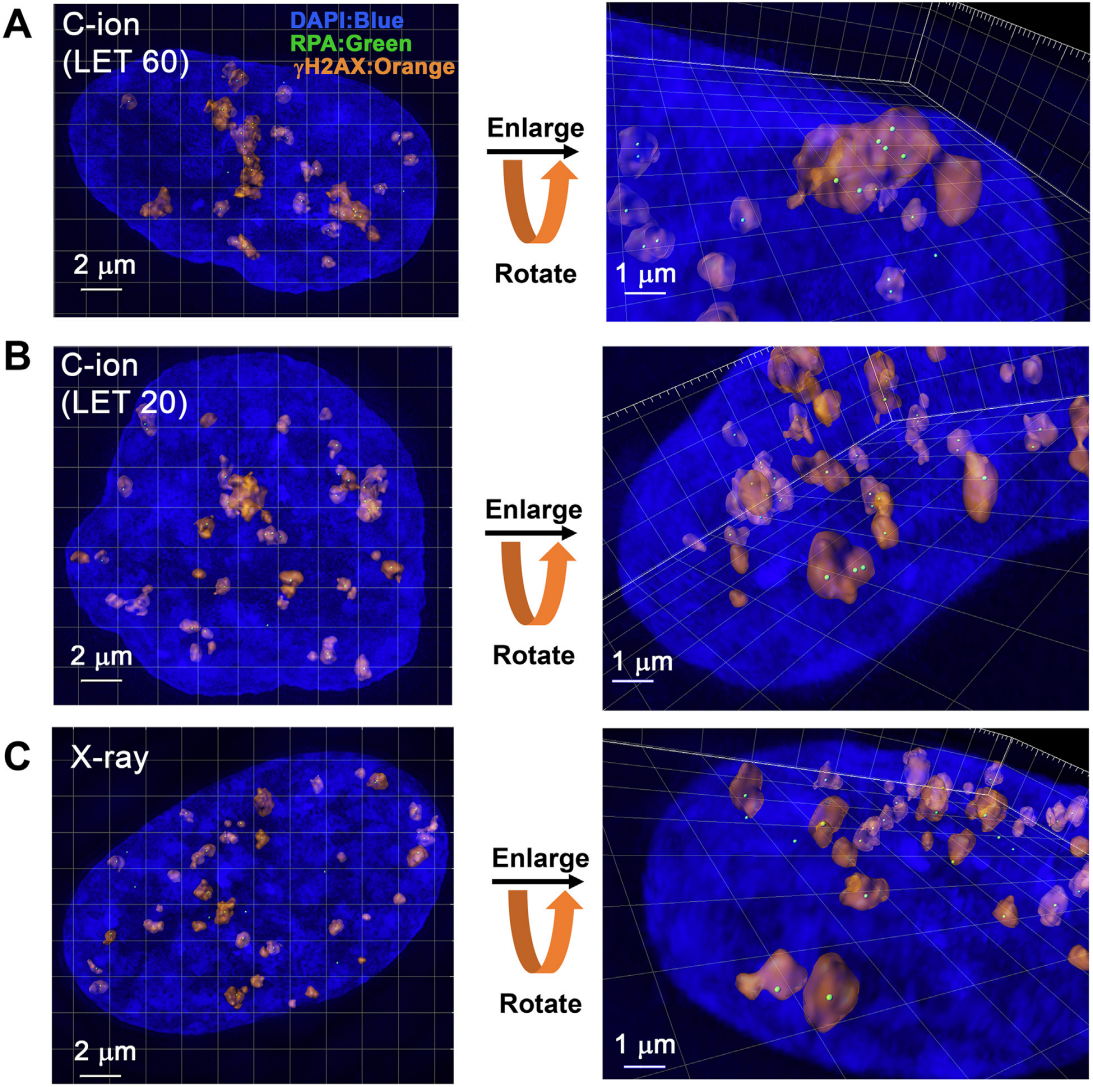
3D-SIM analysis revealed clustered RPA foci formation in the γH2AX foci in G_2_-phase cells after C-ion irradiation **(A, B)** 1BR hTERT cells were irradiated with 1 Gy C-ion irradiation with LET 60 or 20 keV/μm. Cells were fixed 2 h after irradiation and stained with γH2AX, RPA, CENPF and DAPI. The surface polygon images of γH2AX foci (75% semi-transparent) were generated by Imaris 8.1.2. The centre of the fluorescence intensity of the RPA foci is shown by a green spot. Enlarged images with angles are shown in the right panel. **(C)** 1BR hTERT cells were irradiated with 1 Gy X-rays. Surface polygon images of γH2AX and RPA foci are shown. Enlarged images with angles are shown in the right panel.

**Figure 3:**
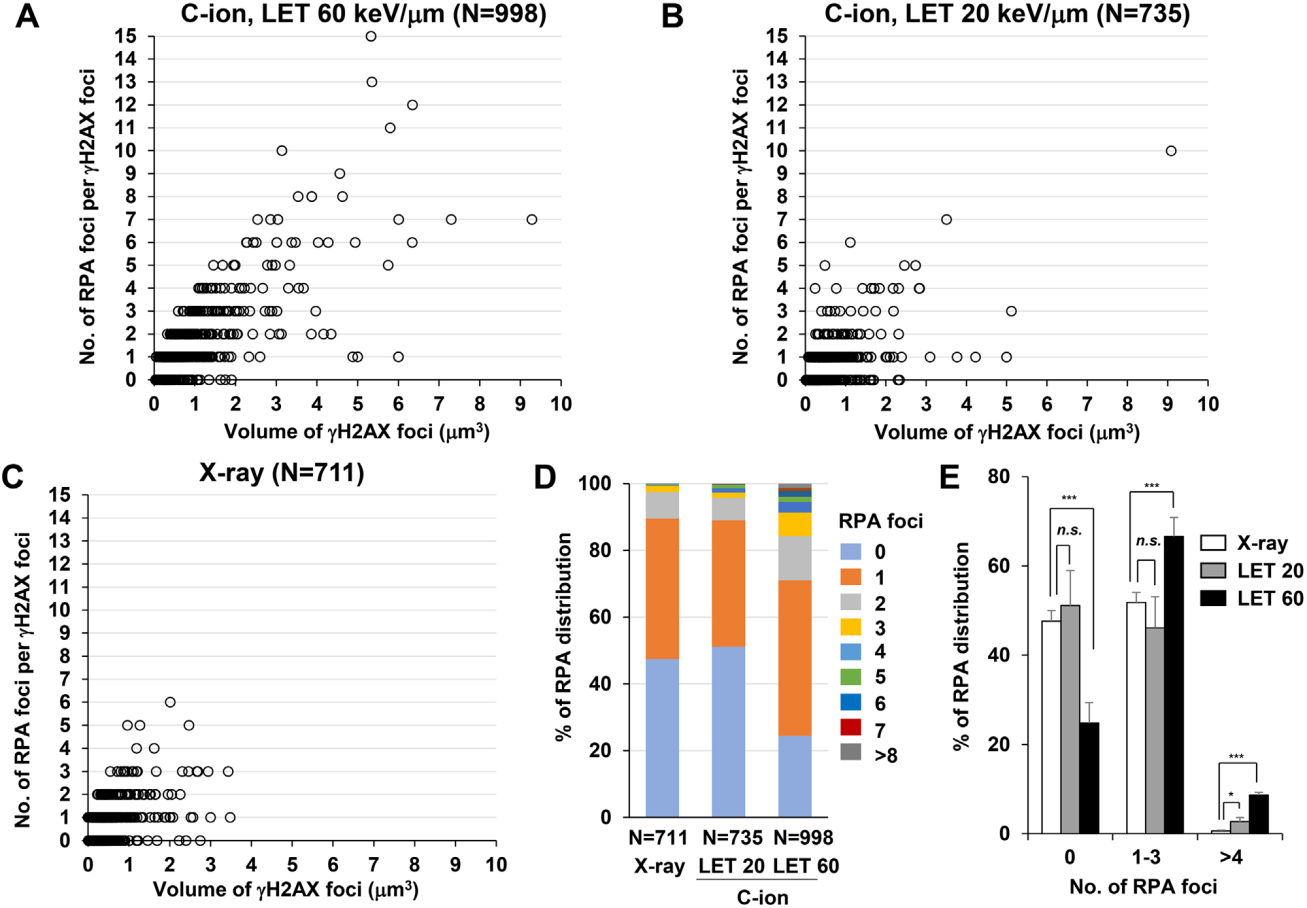
C-ion irradiation causes clustered RPA foci formation in an LET-dependent manner **(A-C)** The number of RPA foci in G_2_ cells was scored in correlation with the volume of γH2AX foci. 1BR hTERT cells were irradiated with 1 Gy C-ion irradiation with LET 60 keV/μm (A), LET 20 keV/μm (B) or 1 Gy X-rays (C). Cells were fixed 2 h after irradiation and stained with γH2AX, RPA, CENPF and DAPI. **(D)** The percentage of γH2AX foci containing different number of RPA foci in 1BR hTERT cells is shown. **(E)** The percentage of γH2AX foci containing different number of RPA foci in 1BR hTERT cells is categorized (0, 1-3 and >4 RPA foci per γH2AX focus) and compared among X-rays, C-ion irradiation with LET 20 and LET 60 keV/μm. Error bars represent SD of three independent experiments.

**Figure 4:**
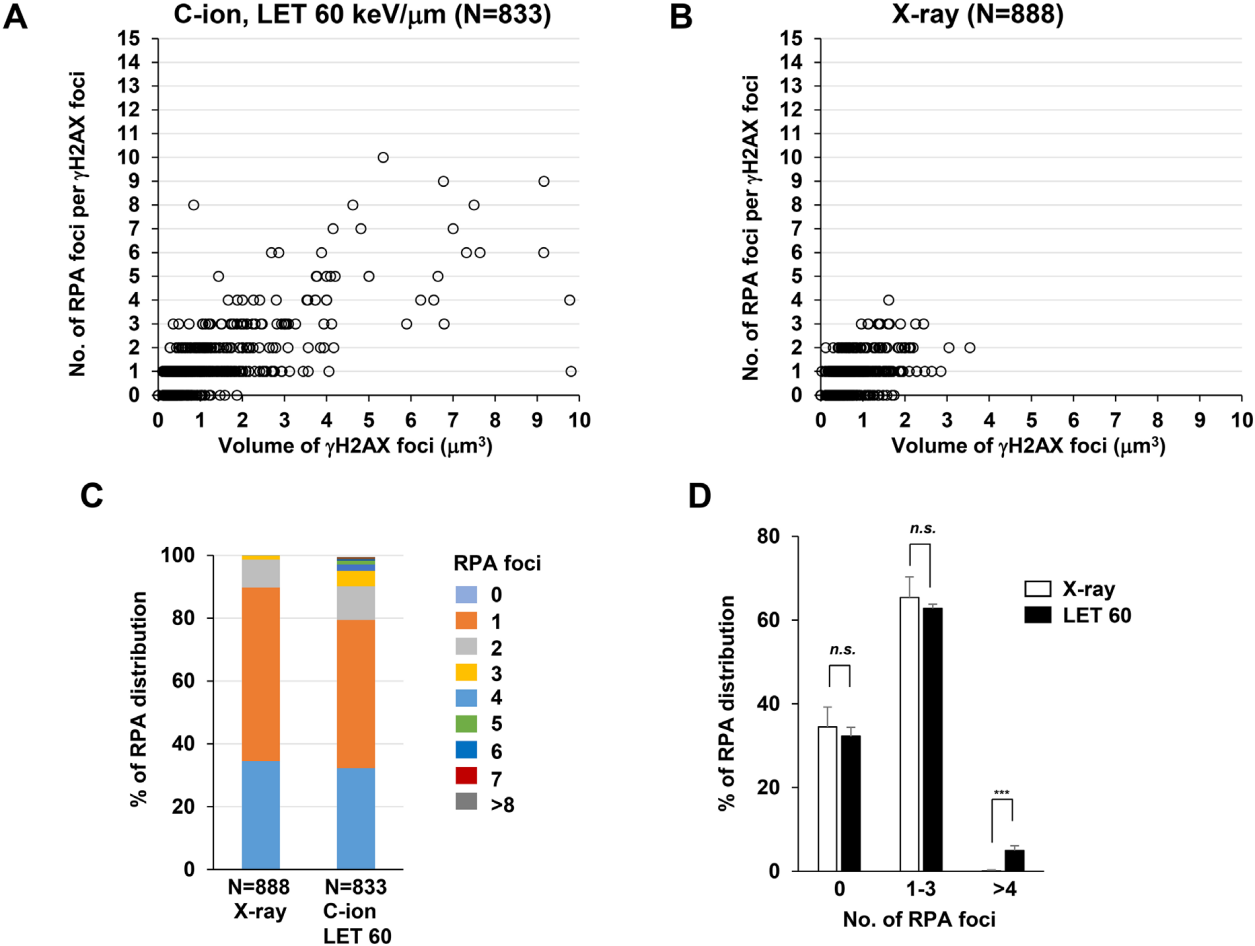
C-ion irradiation causes clustered RPA foci formation in A549 cells **(A-B)** The number of RPA foci in G_2_ cells was scored in correlation with the volume of γH2AX foci. A549 cells were irradiated with 1 Gy C-ion irradiation with LET 60 keV/μm (A) or 1 Gy X-rays (B). Cells were fixed 2 h after irradiation and stained with γH2AX, RPA, CENPF, and DAPI. **(C)** The percentage of γH2AX foci containing different number of RPA foci in A549 cells is shown. **(D)** The percentage of γH2AX foci containing different number of RPA foci in A549 cells is categorized as described in Figure 3E. Error bars represent SD of three independent experiments.

Next, to identify the site of DSBs in γH2AX foci, cells were stained with RPA after IR. In HR, RPA is bound to single-stranded DNA (ssDNA) after DSB end resection, followed by the recruitment of RAD51 [1]. Therefore, resection defects impair RAD51 loading and HR [28]. Because the HR pathway becomes active in cells during the S/G_2_-phase, we analysed irradiated G_2_-cells, which were identified by CENPF (Supplementary Figure 1; Note: because replicating cells contain not only two-ended DSBs but also DNA replication-associated DSBs, cells in S phase were excluded in this study) [16]. To investigate the spatial distribution of RPA foci within the γH2AX signal, sphere spots indicating the centre of RPA foci fluorescence intensity were generated by polygon rendering (Figure 2A-2C, Supplementary Figure 5 and Supplementary Movie 1). Interestingly, we found that multiple RPA foci were formed within γH2AX foci 2 h after high LET C-ion irradiation, whereas clustered RPA foci were rarely observed after X-rays (Figure 2A-2C, Figure 3A-3E and the percentage of Figure 3B is shown in Supplementary Table 1). Importantly, clustering levels after low LET (20 keV/μm) C-ion irradiation were lower than after high LET (60 keV/μm) C-ion irradiation, suggesting that LET is an important factor for the formation of clustered DSBs. However, it should be stressed that, although high LET C-ion irradiation induced clustered RPA foci formation, approximately 70% of γH2AX foci encompass less than 3 RPA foci (Figure 3D, 3E). Next, the clustering levels of RPA foci within γH2AX foci were examined in A549 cells (a lung cancer cell line). The 3D-SIM analysis revealed that high LET C-ion irradiation induced a greater clustered RPA foci formation than X-rays in A549 cells (Figure 4A-4C). Furthermore, similar to the result in 1BR hTERT cells, the percentage of >4 RPA foci within γH2AX foci was statistically greater than that after X-rays in A549 cells (Figure 4D). These results suggest that C-ion irradiation induces clustered DSB formation, although clustered DSBs are not the major population of γH2AX foci even after C-ion irradiation (20-60 keV/μm).

### γH2AX foci volume increases in correlation with the number of RPA foci

**Figure 5:**
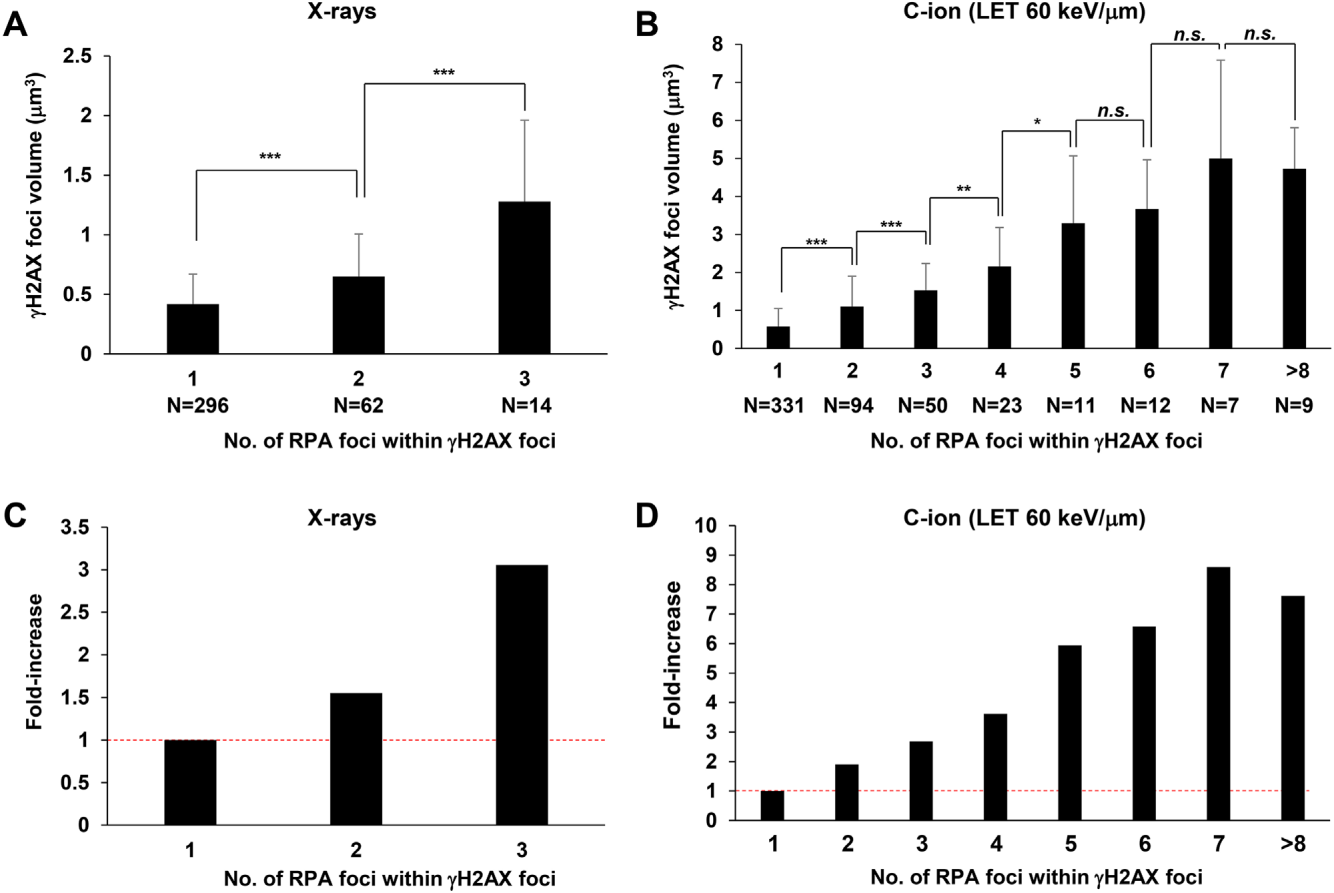
Increase in γH2AX foci volume is correlated with the number of RPA foci **(A, B)** The volume of γH2AX foci in irradiated 1BR hTERT cells was measured when the γH2AX foci contained 1–3 RPA foci after 1 Gy X-rays (A) or 1 to >8 foci after 1 Gy C-ion irradiation with LET 60 keV/μm (B). Cells were fixed 2 h after irradiation and stained with γH2AX, RPA, CENPF and DAPI. **(C, D)** Fold increase in the volume of γH2AX in 1BR hTERT cells is shown. Error bars represent SD of the analysed sample.

**Figure 6:**
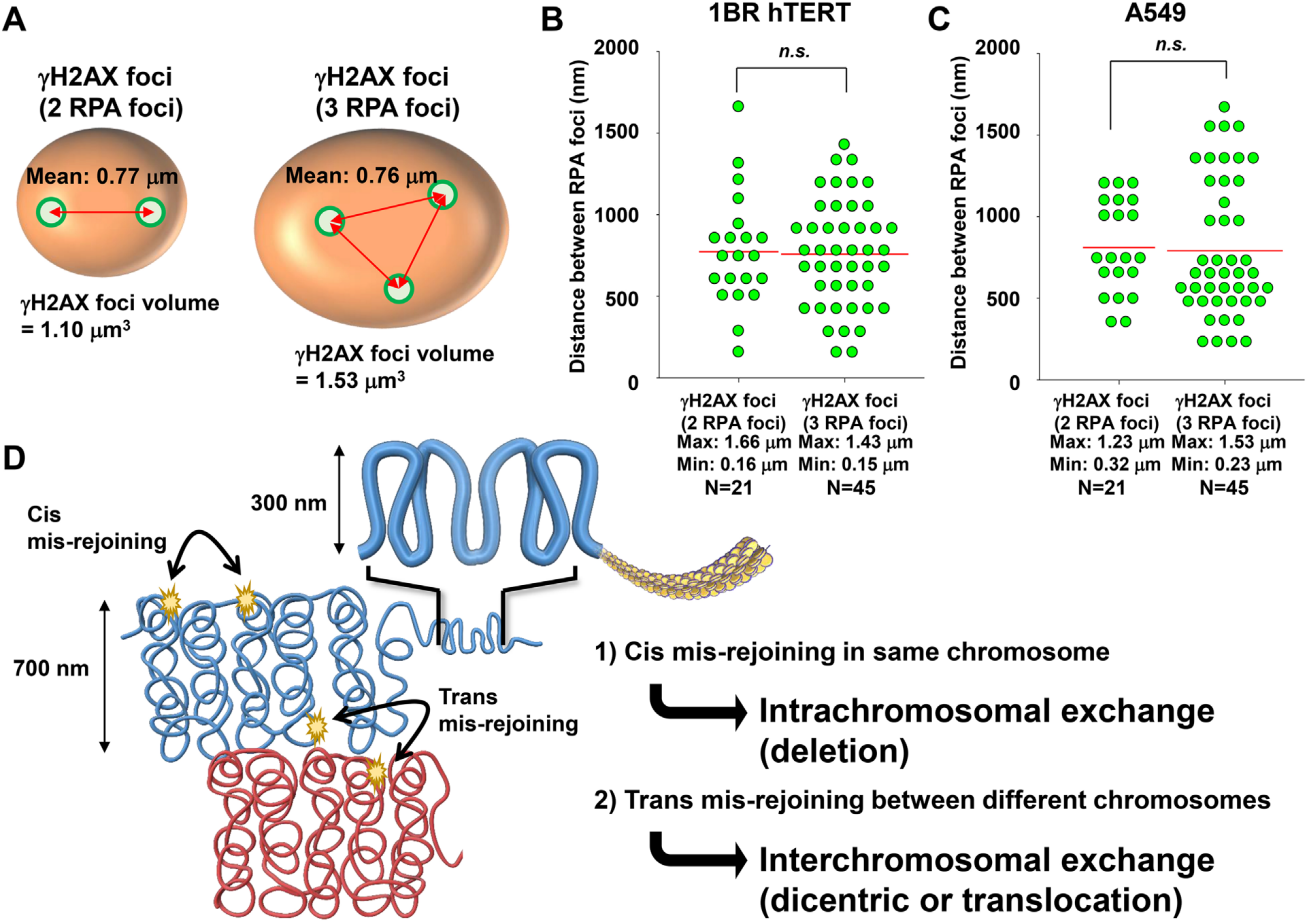
Distribution of RPA foci within γH2AX foci **(A, B)** The distance of two RPA foci within γH2AX foci was measured when the γH2AX foci contained either two or three RPA foci. After 1 Gy C-ion irradiation (LET 60 keV/μm), 1BR hTERT cells were fixed 2 h after irradiation and stained with γH2AX, RPA, CENPF and DAPI. **(C)** The distance of two RPA foci within γH2AX foci in A549 cells is shown. After 1 Gy C-ion irradiation (LET 60 keV/μm), A549 cells were fixed 2 h after irradiation and stained with γH2AX, RPA, CENPF, and DAPI. **(D)** Model for the formation of chromosomal rearrangements after high LET heavy-ion particle irradiation. Following the formation of clustered DSBs in close proximity on the order of a several hundred nanometres, if two DSBs are formed closely in the same chromosome, then intrachromosomal exchange such as a deletion may occur. If two DSBs are formed closely at the chromosome boundary between different chromosomes, then interchromosomal exchange such as a dicentric or translocation may occur.

Next, to address whether these multiple RPA foci are related to the widespread γH2AX foci after C-ion irradiation, we examined the correlation between the volume of γH2AX foci and the number of RPA foci. γH2AX foci encompassing a single RPA focus showed an approximate volume of 0.5 μm^3^ after IR (Figure 5A, 5B). Importantly, we found that an increase in γH2AX foci volume was correlated with the number of RPA foci, although the increase seems to saturate when the γH2AX foci encompass more than 5 RPA foci (Figure 5A-5D). Finally, to examine the spatial distance between any two individual RPA foci within the γH2AX foci, the distance in γH2AX foci encompassing 2 or 3 RPA foci was measured in 3D. The average distance was approximately 700 nm (range, 150–1500 nm) (Figure 6A, 6B). A similar distribution was observed in A549 cells (Figure 6C). Interestingly, the distance was similar even if the γH2AX foci encompassed 2 or 3 RPA foci (Figure 6B, 6C). Although it is unclear whether two DSBs influence the expansion of H2AX phosphorylation, our data suggest that γH2AX foci can spread to an almost double volume if at least two DSBs are located within an approximate distance of 700 nm of each other. Taken together, these findings strongly suggest that clustered DSBs in close proximity, on a scale of approximately 1 μm^3^, are formed along the track after high LET particle irradiation.

## DISCUSSION

The formation of >2 DSBs in close proximity can be a risk for deletion if DSBs occur in the same chromosome, and also for interchromosomal exchange, such as a dicentric and translocation if DSBs occur in two distinct chromosomes (Figure 6D). A recent study demonstrated that C-ion irradiation induced DSBs were eventually repaired, even though an increased RBE was observed for cell survival [29]. Thus, misrepair resulting in lethal chromosomal rearrangements, such as a dicentirc or a translocation/deletion within an essential gene, can be a critical factor leading to the increased RBE of heavy-ion radiation. We previously demonstrated that heavy-ion particle radiation frequently induces γH2AX foci at chromosome boundaries by using γH2AX-chromosome FISH analysis [30]. Based on the contact first model, which suggests the joining of two broken chromosomes takes place when the breaks are located in a proximal position [31], if the clustered DSBs occur at the chromosome boundary, it will likely lead to interchromosomal exchange [32]. Consistently, previous studies have shown that high LET particle radiation causes a high frequency of chromosomal aberrations compared with that observed after X-rays, even if it is low dose [33]. We previously showed a variable length and number of tracks between individual adherent cells after horizontal particle irradiation [17]. The uneven distribution was likely caused because the plane of individual cells differs and the particles can traverse with different directions, even though theoretically equal distribution of particle tracks is expected by a Poisson model in water. For example, although the dose to a cell population is 1 Gy, the dose to any individual cell within the population can be distinct. Hence, our previous findings and those from this study indicate that overall IR doses to a cell population are not simply correlated with the formation of chromosomal aberrations after particle irradiation [34], but, rather, the number and separation of particle traversals through each individual nucleus are critical factors determining the number and severity of chromosomal aberrations. From the point of view of cancer treatment, the levels of clustered DSBs and the track length may be important factors that determine the efficacy of C-ion therapy. Thus, it is important to examine the relationship between clustered DSBs, particle tracks, and cell fate in the future.

Complex DNA lesions are defined as DNA damage encompassing multiple types of damage, including DSBs, SSBs and base damage (oxidised bases or abasic sites), within 1–2 helical turns [13]. This is an important hallmark of DNA lesions after high LET heavy-ion particle radiation. The scale of such complex lesions is estimated at 3–8 nm when the length of one helical turn of DNA is approximately 3–4 nm. Because the resolution of 3D-SIM is >135 nm, it is unlikely that our 3D-SIM analysis could capture such clustered DNA lesions. RPA is a protein which is loaded onto ssDNA following DSB end resection in HR [1]. Because the length of resection is estimated to be 0.2–1 kbp (i.e. 1–5 nucleosomes) [35], the resected DNA may be compacted in an approximate volume of <0.03 μm × 0.06 μm × 0.06 μm (1.08 × 10^−4^ μm^3^), which contains 4 nucleosomes when one nucleosome and linker are compacted within a square 30 nm each side in chromatin fibre. To verify that RPA foci represent DSBs, we confirmed that the formation of RPA foci within γH2AX foci is CtIP/EXO1-dependent (Supplementary Figure 6)[28]. These data strongly suggest that RPA foci within γH2AX foci are formed by enzyme-dependent resection in DSB repair (i.e., RPA foci represent DSBs undergoing resection during HR). One of the reasons RPA foci formation was used in this study is that the fluorescence intensity of RPA foci is clear because possibly multiple RPA molecules directly bind to ssDNA forming protein-filaments on the DNA. This provides sufficiently strong fluorescence intensity for 3D-SIM analysis. In addition, because RPA binds to ssDNA following resection, the binding to DNA is unlikely affected by the complexity of DNA damage surrounding the DSB end because they are removed during resection. Recently, the visualization of NHEJ factors has been demonstrated by super-resolution microscopy [36, 37]. These studies elegantly revealed the recruitment of 1–2 molecules of NHEJ protein at DSB sites. However, because only a few molecules of NHEJ protein bind to the DSB end, sufficient fluorescence intensity is not always obtained, which may result in underestimation of the absolute number of breaks. Further, it is unknown whether heavy-ion induced DSB ends always allow binding of the NHEJ protein, particularly when there is lesion complexity. A recent study has suggested that base excision repair (BER) completes the repair of complex lesions following the repair of DSB by NHEJ [38], however, it remains unclear how the repair of complex lesions are spatiotemporally coordinated. For example, if a DSB is indirectly induced during BER/SSB repair when two or more base damages/SSBs occur on opposite strands within several base pairs, then BER/SSB repair proteins may block the binding of Ku at the DSB end. We understand that our RPA foci may also underestimate the absolute number of DSBs because all DSB ends may not undergo resection, even though high LET particle radiation efficiently promotes resection compared with X-rays [39]. Also, there may be some underestimation of the absolute number of DSBs in the present study if multiple DSBs are formed on a <135 nm scale. A comparison of the number between RPA foci and DSBs estimated by simulation shows that 1 Gy C-ion irradiation with LET of 60 keV/μm causes 60 DSBs per G_2_ cell (4N) [40], whereas the average of the number of RPA foci per cell was 34.4 in G_2_ cells in this study. This suggests that multiple DSBs are formed in the scale of 100 nm. Nevertheless, our findings strongly suggest that high LET particle radiation can cause clusters containing >2 DSBs in close proximity on a scale of approximately 1 μm^3^ along the track.

In this study, we used C-ion irradiation with LET of 20 or 60 keV/μm, which is in the range of C-ion therapy. We showed that C-ion irradiation caused clustered RPA foci in 1BR hTERT human fibroblasts and A549 cancer cells. The data showed that the level of clustered DSBs in A549 cells was slightly less than 1BR hTERT cells and the volume of γH2AX foci seems greater than in 1BR hTERT cells. In addition, the distribution of the distance between RPA foci in A549 cells is greater than that in 1BR hTERT cells. These distinctions between normal fibroblast and cancer cells might be caused by a variation of chromatin compaction [41, 42]. LET is an important parameter of DNA damage after heavy-ion particle irradiation. Although we examined the clustering of DSBs in the range of C-ion therapy, it would be interesting to address the magnitude of clustering after >100-200 keV/μm, which may induce highly condensed clustered DSBs on the scale of 1 μm^3^ in future work. Interestingly, we found that X-rays also induce 2–3 RPA foci within γH2AX foci although it is significantly less than after C-ion irradiation. This observation may be supported by the observation that low LET X-rays also cause complex DNA lesions [43], although clearly, the magnitude of cluster formation is less than with high LET radiation.

In the present study, 3D-SIM analysis revealed clustered DSB formation within a scale of approximately 1 μm^3^ after high LET particle irradiation. This clustered DSB formation may be the cause of formation of short DNA fragments, which is another hallmark of heavy-ion irradiation [44]. In addition to the formation of complex DNA lesions including DSBs, SSBs and base damage, clustered DSBs should be considered as another hallmark of high LET heavy-ion radiation. Such clustered DSBs may underlie the cancer cell-killing effects of heavy-ion radiotherapy, likely contributing to the formation of chromosomal rearrangement, and leading to cell death.

## MATERIALS AND METHODS

### Cell culture, irradiation and drug treatment

1BR (human fibroblasts) hTERT cells or A549 cells were cultured in the Alpha modification of minimum essential medium (MEM) (Wako, Osaka, Japan) or Eagle's MEM with 10% fetal calf serum (Sigma-Aldrich, St. Louis, MO, USA), respectively. X-ray irradiation was performed using a Faxitron RX-650 (100 kVp, 1.14 Gy/min, Faxitron Bioptics, Tucson, AZ, USA) with a dose rate of approximately 1 Gy/min. C-ion irradiation was performed at Gunma University Heavy Ion Medical Center; GHMC (290 MeV/n, LET 20 or 60 keV/μm) [45]. The LET value at the irradiation position was derived using Monte Carlo simulations. The position of the Bragg peak in the physical dose distribution was measured using an ionisation chamber at depth in a water phantom. The depth in the water phantom was adjusted so that the cells were placed 2.2 mm before the Bragg peak to set up 60 keV/μm. To visualize the particle track, horizontal irradiations were conducted with a mono-energetic broad beam.

### Immunofluorescence staining

1BR hTERT cells were seeded on Thermo Scientific™ Nunc™ Lab-Tek™ II Chambered Coverglass (Thermo, Rochester, NY, USA) 2 days before the experiment to obtain exponentially growing cells. G_2_ cells were identified by CENPF staining [16]. To prevent cell cycle progression from S to G_2_ during analysis, aphidicolin (APH; Wako, Osaka, Japan) was added 30 min prior to irradiation (Supplementary Figure 1)[16]. The pan-nuclear γH2AX signal in S-phase cells is enriched by the treatment with APH (Supplementary Figure 1)[16, 20]. At the indicated time-points, cells were treated with 0.2% TritonX-100-PBS (Phosphate Buffered Saline) for 1 min, then fixed with 3% paraformaldehyde-2% Sucrose for 10 min. Following a 1x PBS wash, cells were incubated for 30 min at 37°C with primary antibody in 2% BSA-PBS. Cells were then washed with PBS and incubated with appropriate secondary antibodies conjugated to Alexa Fluor 488/594/647 in 2% BSA-PBS including 0.1 mg/mL 4’,6-Diamidino-2-Phenylindole, dihydrochloride (DAPI; Roche, Mannheim, Germany) for 30 min at 37°C. After washing in PBS, slides were mounted in Vectashield (Vector Laboratories, Burlingame, CA, USA). Antibodies are listed in Supplementary Table 2.

### Acquisition of 3D-SIM image

3D-SIM was performed on a microscope system (DeltaVision OMX version 4, GE Healthcare UK Ltd) equipped with 405, 488 and 568 nm solid-state lasers. Images were acquired using a Plan Apo N × 60, 1.42 NA oil immersion objective lens (Olympus, Tokyo, Japan) and one liquid-cooled sCMOs cameras (PCO, Kelheim, Germany). Exciting light was directed through a movable optical grating to generate a fine-striped interference pattern on the sample plane. The pattern was shifted laterally through five phases and three angular rotations of 60° for each z section. Optical z-sections were separated by 0.125 μm. The laser lines 405, 488 and 568 nm were used for 3D-SIM acquisition. Exposure times were typically between 60 and 80 ms, and the power of each laser was adjusted to achieve optimal intensities of between 4,000 and 15,000 counts in a raw image of 15-bit dynamic range at the lowest laser power possible to minimize photobleaching. Multichannel imaging was achieved through sequential acquisition of wavelengths by separate cameras. Raw 3D-SIM images were processed and reconstructed using the DeltaVision OMX SoftWoRx 6.1 software package (GE Healthcare). The lateral and axial resolutions of 3D-SIM images were >135 ± 5 nm and >350 ± 15 nm, respectively. The details of resolution are described on the GE Healthcare website (https://www.gelifesciences.com/gehcls_images/GELS/Related%20Content/Files/1407240581290/litdoc29115193_20161015171810.pdf). The resulting size of the reconstructed images was of 1024 × 1024 pixels from an initial set of 512 × 512 raw images. The channels were then carefully aligned using alignment parameters from control measurements with Image registration calibration slide and 0.1 μm TetraSpeck™ Fluorescent Microspheres (Molecular Probes, Eugene, OR, USA).

### Analysis of γH2AX and RPA foci and statistical analysis

Three dimensional γH2AX polygon rendering was generated by surface mode in Imaris 8.1.2 (Bitplane, Zurich, Switzerland). The threshold for generating polygon rendering is applied following the automatic setting using the 0.1 μm surface setting in Imaris 8.1.2. Following polygon rendering, the three dimensional mean volume of bounding box (Axis-Aligned: AA), bounding box (Object-Oriented: OO) and γH2AX object volume were measured by Imaris 8.1.2. The centre of fluorescence intensity of the RPA foci was identified by spot mode and visualized as a green spot in Imaris 8.1.2. The numbers of RPA foci within 75% semi-transparent γH2AX signals were scored by eye on the Imaris 8.1.2 screen in 3D. The distance between RPA foci was calculated following measurement of the position of the X, Y and Z-axis in 3D. Statistical significance was determined using Student’s two-tailed t test or Mann–Whitney U test by SigmaPlot 12.0. ^*^: *P*<0.05, ^**^: *P*<0.01, ^***^: *P*<0.001. The n.s. represents non significance.

## SUPPLEMENTARY MATERIALS FIGURES, TABLES AND VIDEO




