# Supplementary material for: 3D-structured illumination microscopy reveals clustered DNA double-strand break formation in widespread γH2AX foci after high LET heavy-ion particle radiation
Source: Oncotarget. 2017 Nov 25;8(65):109370–81. doi: 10.18632/oncotarget.22679 (PMC5752527; doi:10.18632/oncotarget.22679)
Supplement: Supplementary file 1 [file oncotarget-08-109370-s001.pdf]

3D-structured illumination microscopy reveals clustered DNA double-strand break formation in widespread γH2AX foci after high LET heavy-ion particle radiation

SUPPLEMENTARY MATERIALS

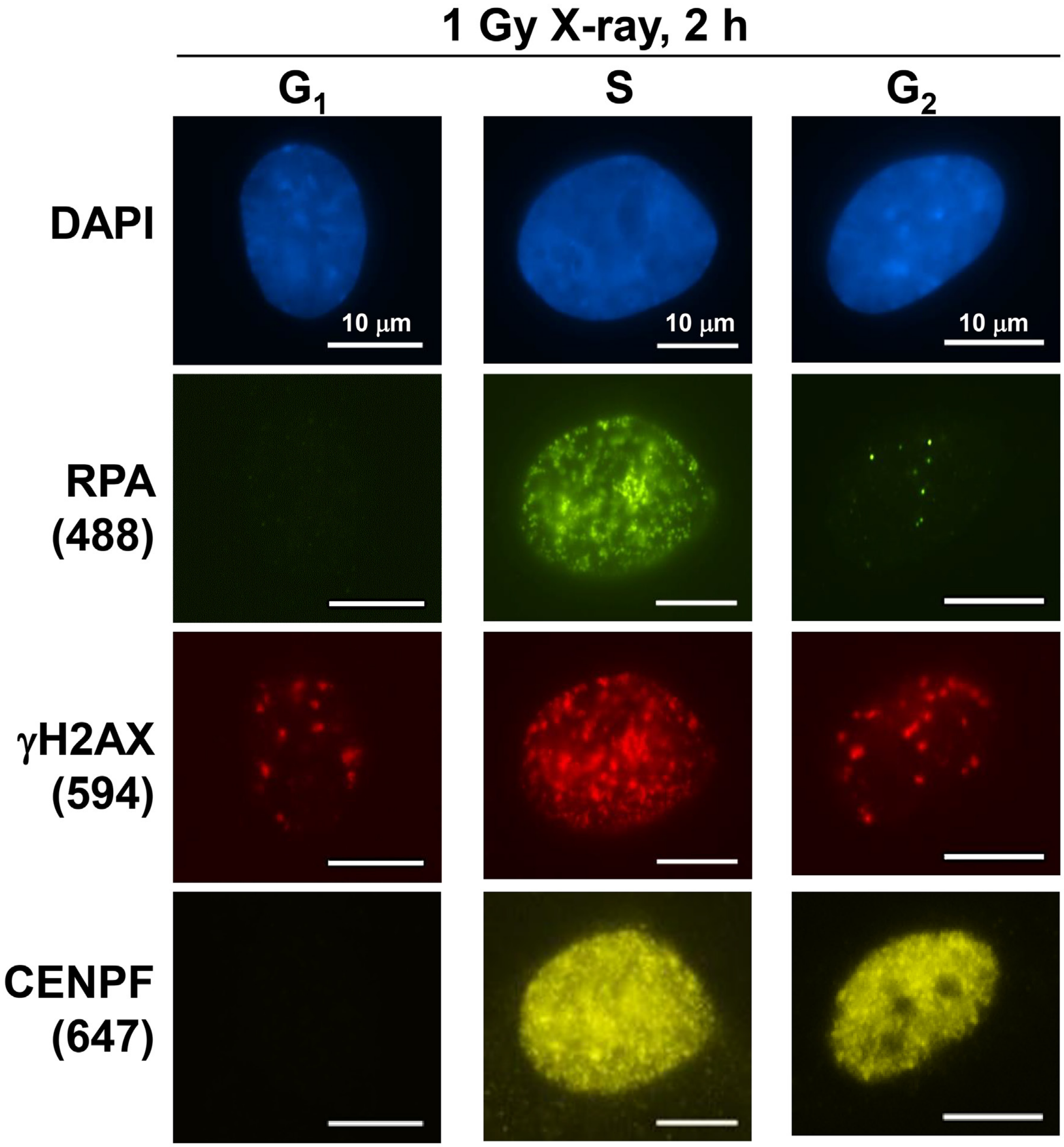

Supplementary Figure 1: Identification of irradiated G_2 cells using CENPF. 1BR hTERT cells were stained with RPA, γH2AX, and CENPF antibodies. G_1 cells were identified as CNEPF− and γH2AX foci+ (not pan-nuclear γH2AX). S cells were identified as CENPF+ and pan-nuclear γH2AX. G_2 cells were identified as CENPF+ and γH2AX foci+ (not pan-nuclear γH2AX).

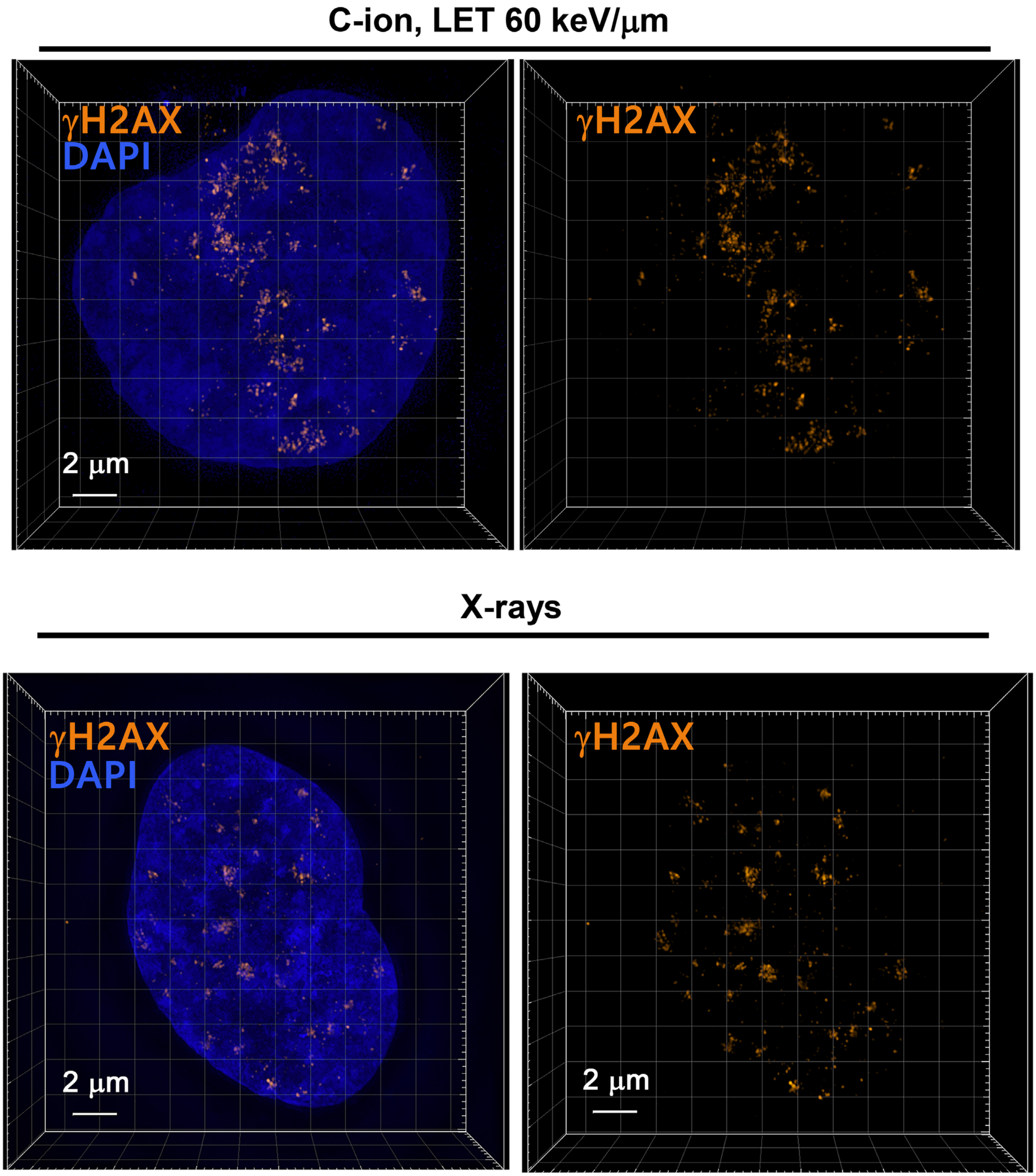

Supplementary Figure 2: Raw image for Figure 1. The raw image of Figure 1 is shown.

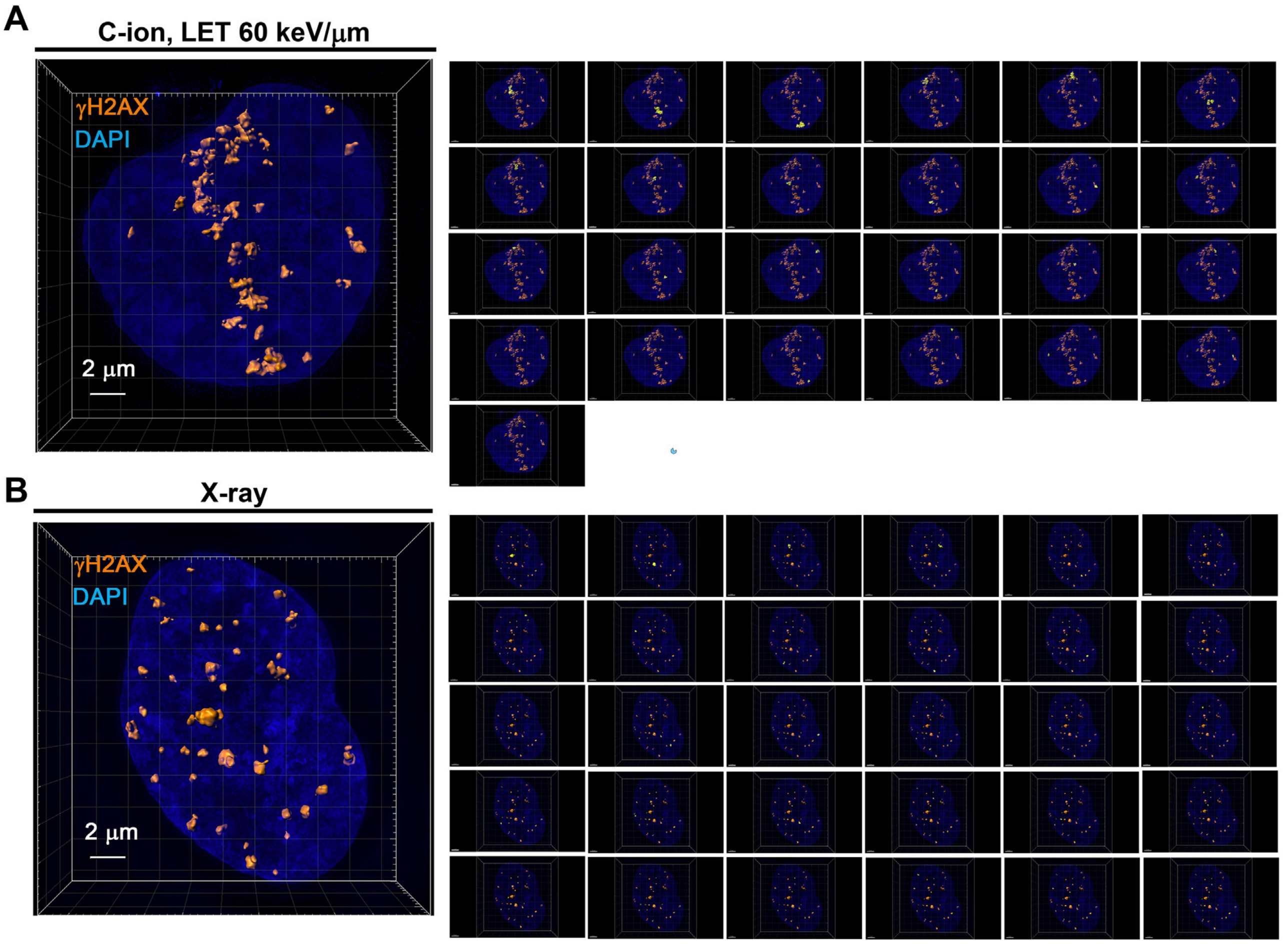

Supplementary Figure 3: Identification of individual γH2AX foci along the track. Individual γH2AX signals after C-ion **(A)** or X-ray irradiation **(B)** were identified following polygon rendering using Imaris 8.1.2. Each γH2AX focus is highlighted in yellow.

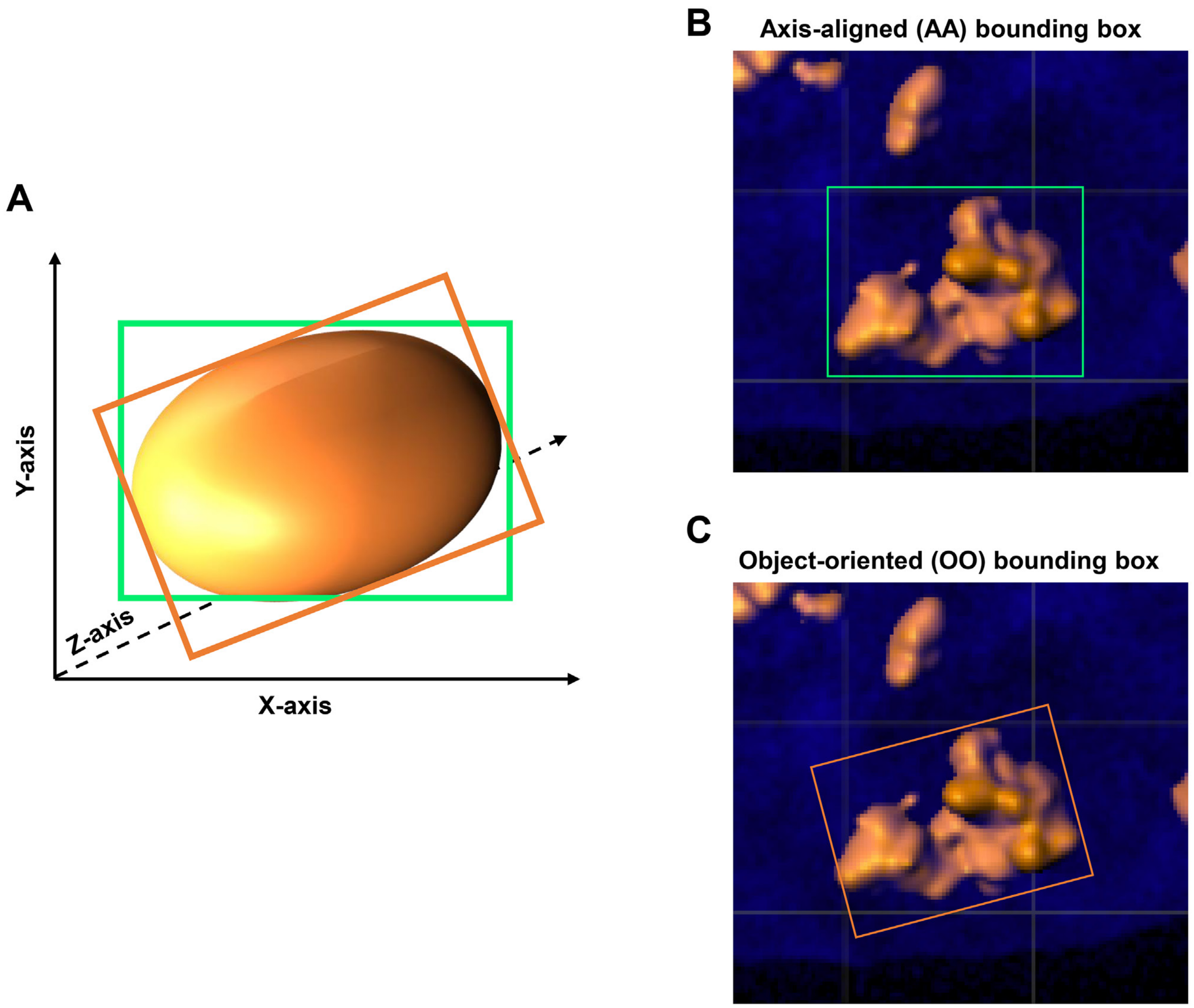

Supplementary Figure 4: Analysis of the axis-aligned (AA) and object-oriented (OO) bounding box. **(A)** Representative modeling of the AA and OO bounding box is shown. **(B, C)** Representative image of the AA and OO bounding box in the x-y axis is shown.

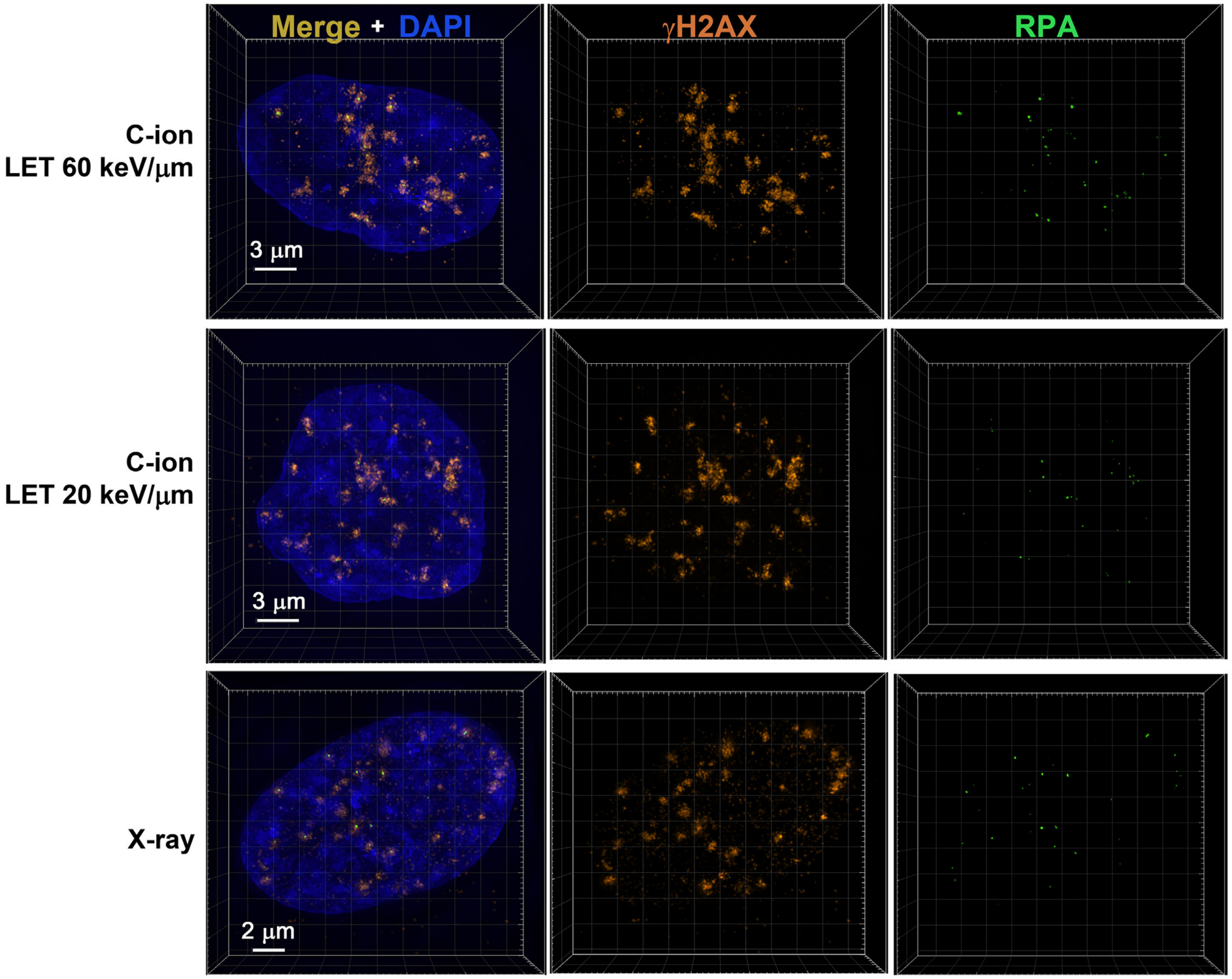

Supplementary Figure 5: Raw image for Figure 2. The raw image of Figure 2 is shown.

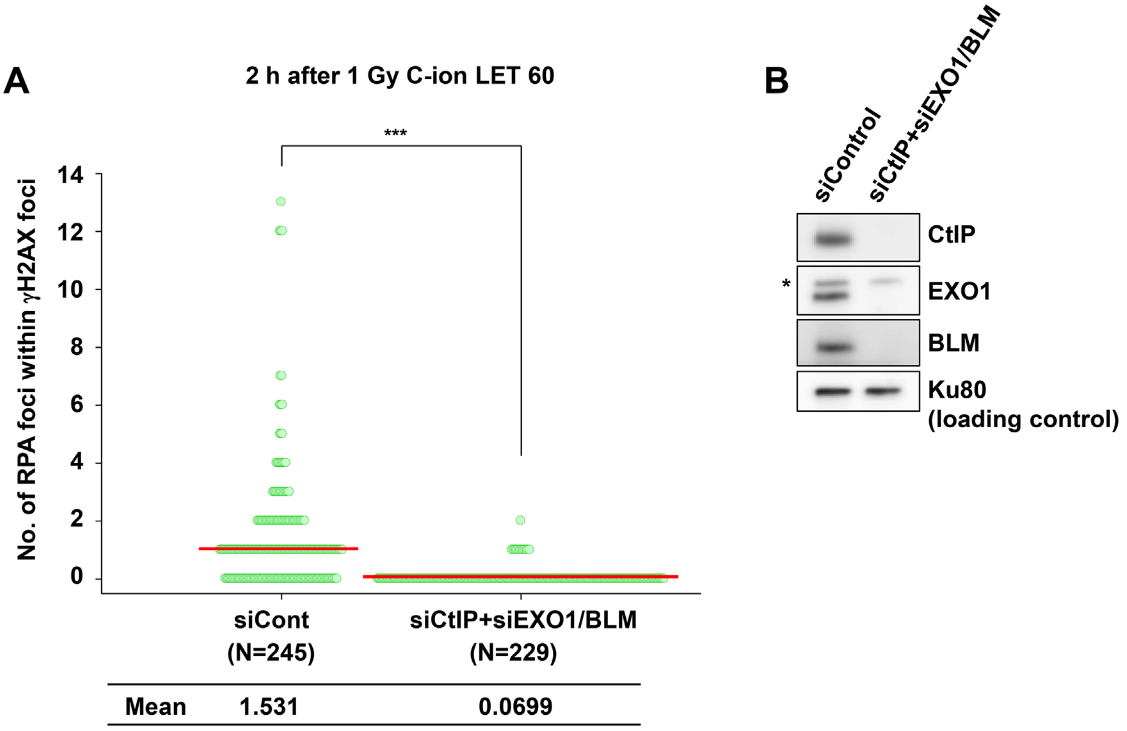

Supplementary Figure 6: RPA foci within γH2AX foci require CtIP/EXO1/BLM-dependent DNA end resection. (A) siRNA transfection of 1BR hTERT cells was performed using Lipofectamine™ RNAiMAX Transfection Reagent (Thermo, Rochester, NY, USA). ON-TARGETplus siRNA (20 µM, Dharmacon) was added to suspended cells after trypsinization. For the 2nd shot of siRNA, cells were re-transfected with siRNA in suspended cells after trypsinization at 24 h after 1st transfection. Cells were incubated for 48 h after the second transfection before IR. RPA foci within γH2AX foci were examined using the SIM image on the screen of Imaris 8.1.2 **(B)** Knockdown efficiency was confirmed by immunoblotting. The asterisk represents a non-specific signal. Ku80 was used as the loading control.

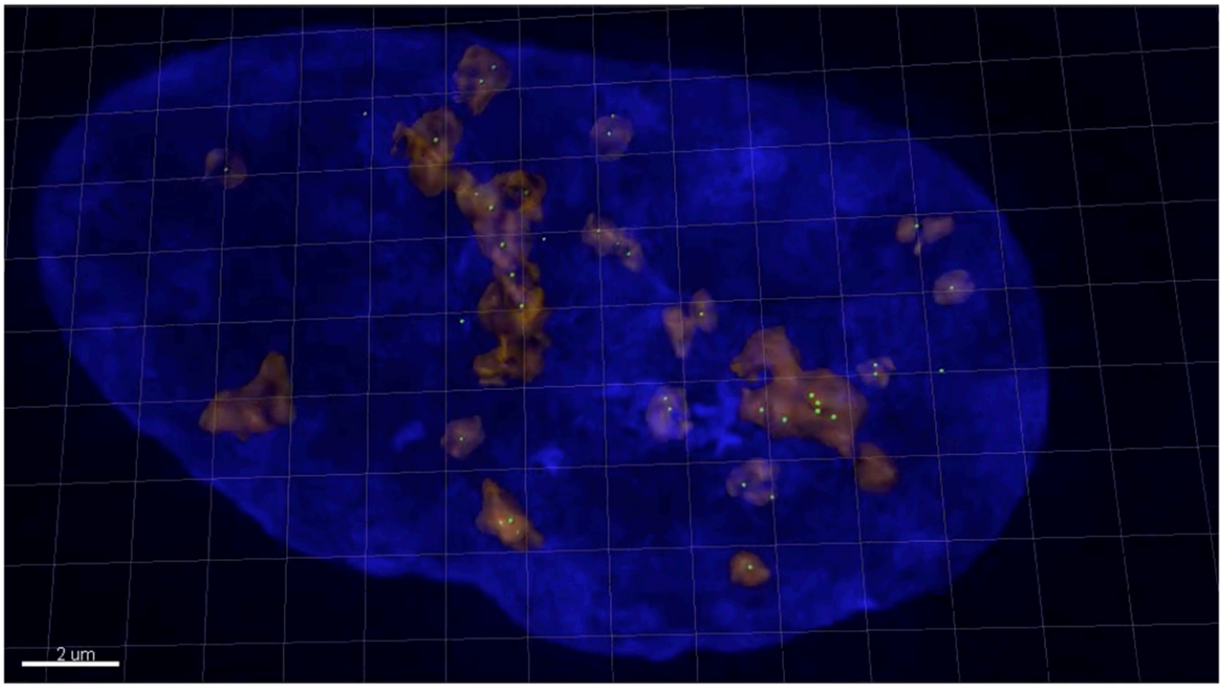

Supplementary Movie 1: The movie of cells with RPA and γH2AX polygon is shown. 1BR hTERT cells were fixed 2 h after 1 Gy C-ion irradiation with LET 60 keV/μm. Cells were stained with RPA, γH2AX, and CENPF antibodies.

Supplementary Table 1: The percentage of RPA foci number in γH2AX foci at 2 h after IR

No of RPA foci with γH2AX foci	C-ion LET 60 (%)	C-ion LET 20 (%)	X-ray (%)
0	24.5	51.2	47.5
1	46.6	37.8	42
2	13.2	6.8	8
3	7.0	1.5	1.9
4	3.2	1.4	0.2
5	1.5	1.0	0.3
6	1.7	0.1	0.1
7	1.0	0.1	0
>8	1.3	0.1	0

Supplementary Table 2: List of antibodies used in the present study

Target	Mono/polyclonal	Clone/reference	Antibody raised in	Source	Dilution for I.F. or I.B.
BLM	Poly	Ab2179	Rabbit	Abcam	1:1000 (I.B.)
CENPF	Poly	Ab5	Rabbit	Abcam	1:1000 (I.F.)
CtIP	Mono	D76F7	Rabbit	Cell Signaling Technology	1:1000 (I.B.)
EXO1	Poly	A302-640A	Rabbit	Bethyl Laboratories, Inc.	1:1000 (I.B.)
γH2AX	Mono	JBW301	Mouse	Upstate Biotechnology	1:800 (I.F.)
Ku80	Mono	C48E7	Rabbit	Cell Signaling Technology	1:1000 (I.B.)
RPA32	Mono	LS-C38952	Rat	Life Span BioSciences, Inc.	1:1000 (I.F.)

I.B., immunoblotting; I.F., Immunofluorescence.
